# Supplementary figures and images for: Widespread loss of safe lake ice access in response to a warming climate
Source: PLoS One. 2024 Dec 11;19(12):e0313994. doi: 10.1371/journal.pone.0313994 (PMC11633986; doi:10.1371/journal.pone.0313994)

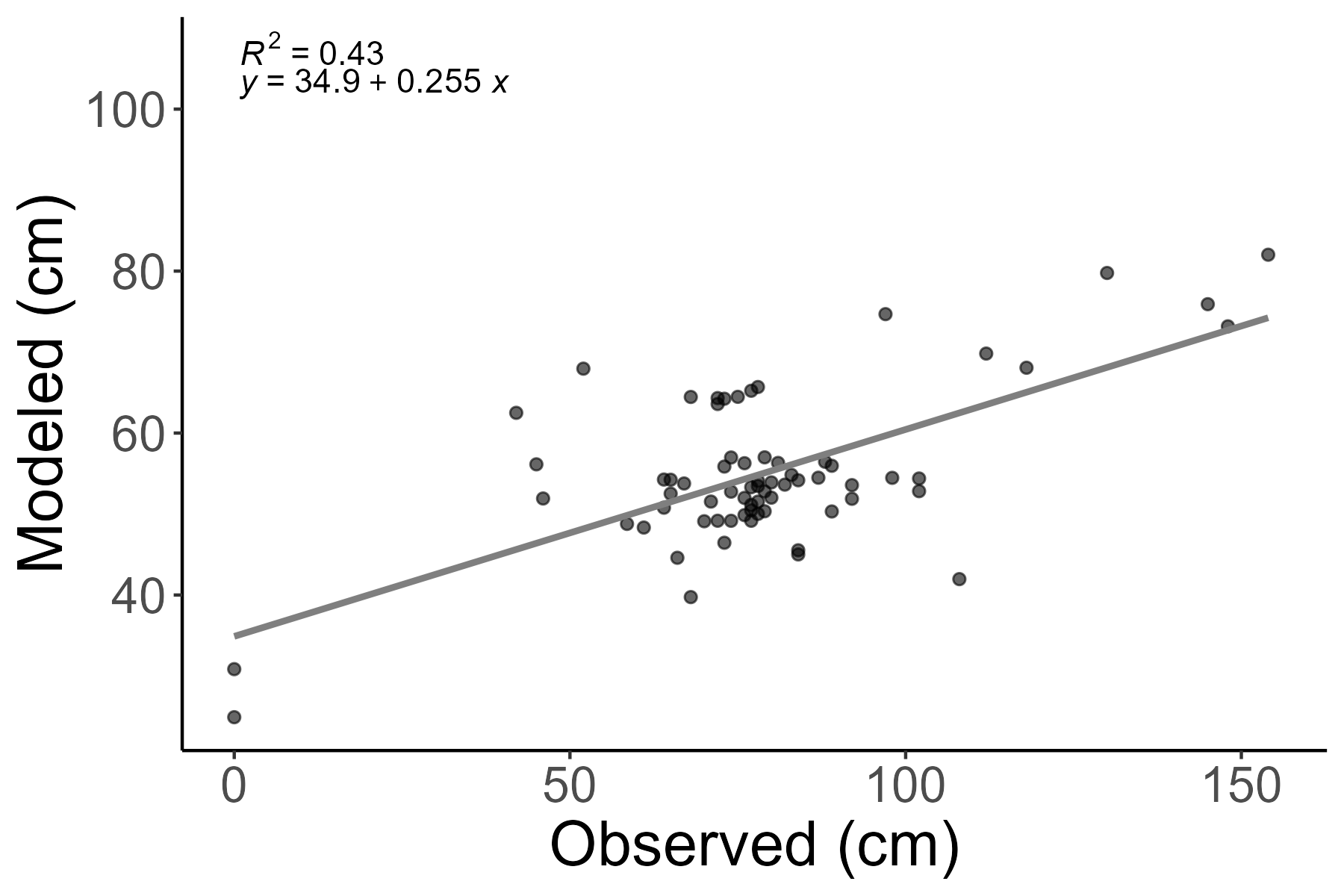

Supplement: S1 Fig — A comparison of modeled and observed data for the maximum ice thickness for each of the 71 validation lakes. Each point represents an individual lake. The line is the regression line with the R2 and line equation in the upper left of the plot. (TIF) [file pone.0313994.s001.tif]

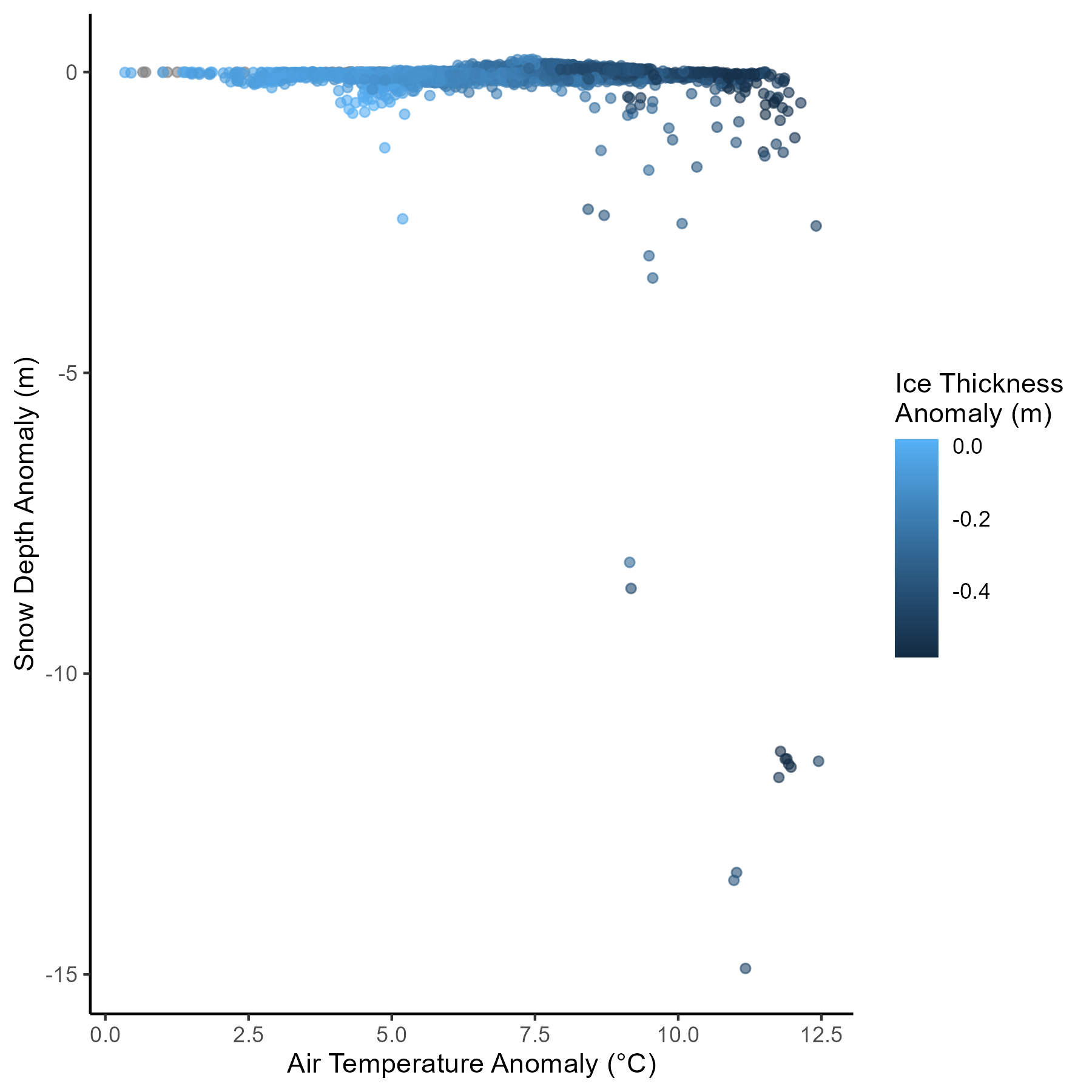

Supplement: S2 Fig — The anomaly of ice thickness comapred to a historical condition (1851–1880) (colored points) when considering the forcing of air temperature anomalies (y-axis) and snow depth anomalies (x-axis). Each point represents the mean of an individual grid cell at 4°C warming. (TIF) [file pone.0313994.s002.tif]

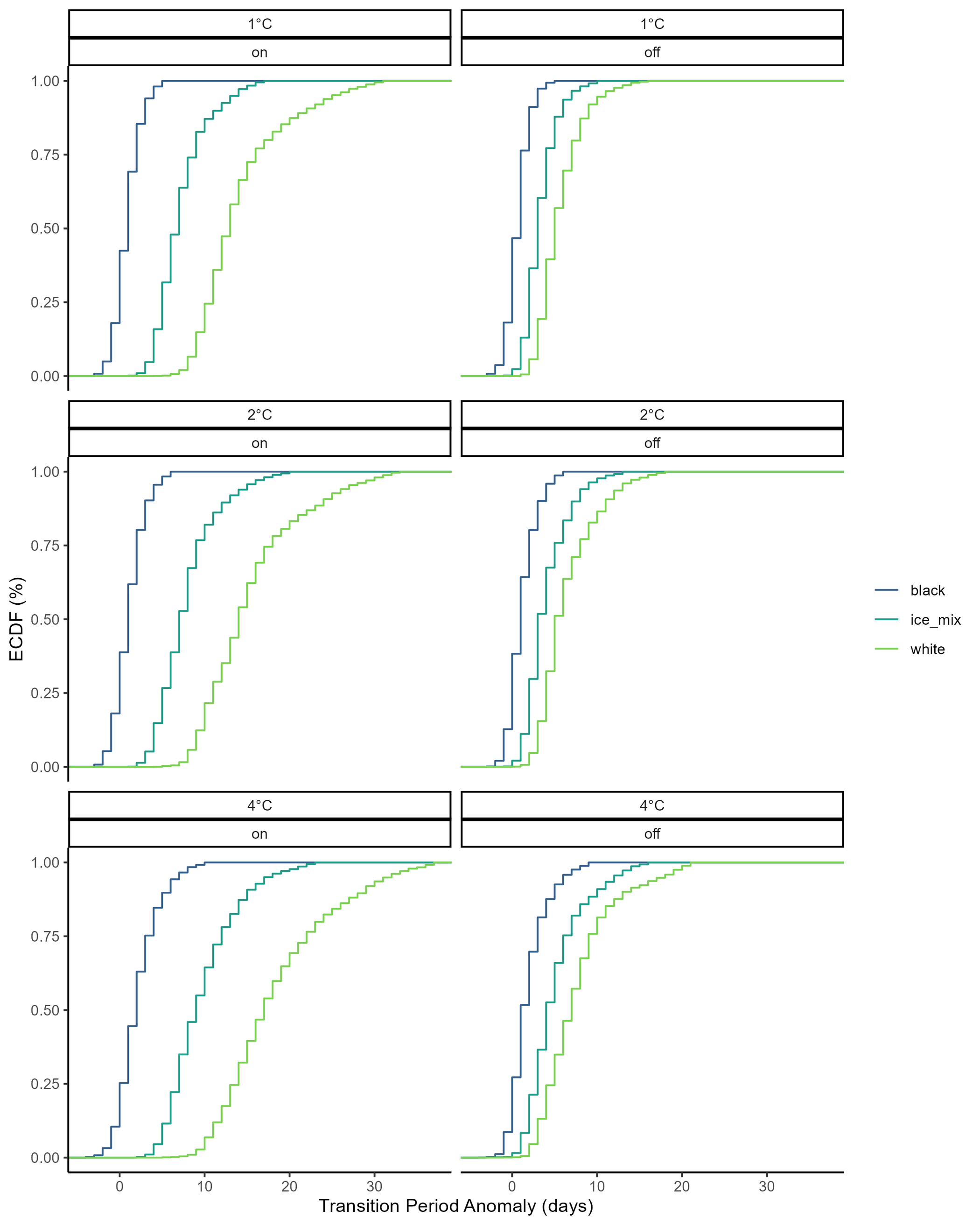

Supplement: S3 Fig — The empirical cumulative distribution functions (ECDF) of each combination of warming scenarios and ice quality scenarios. Represented are (a) the ECDFs of each warming scenario (colored lines for 1°C, 2°C, and 4°C) with ice quality held constant. The left columns of both (a) and (b) show the ice formation transition period (i.e., “on”) and the right columns show the ice melt transition period (i.e., “off”). (TIF) [file pone.0313994.s003.tif]

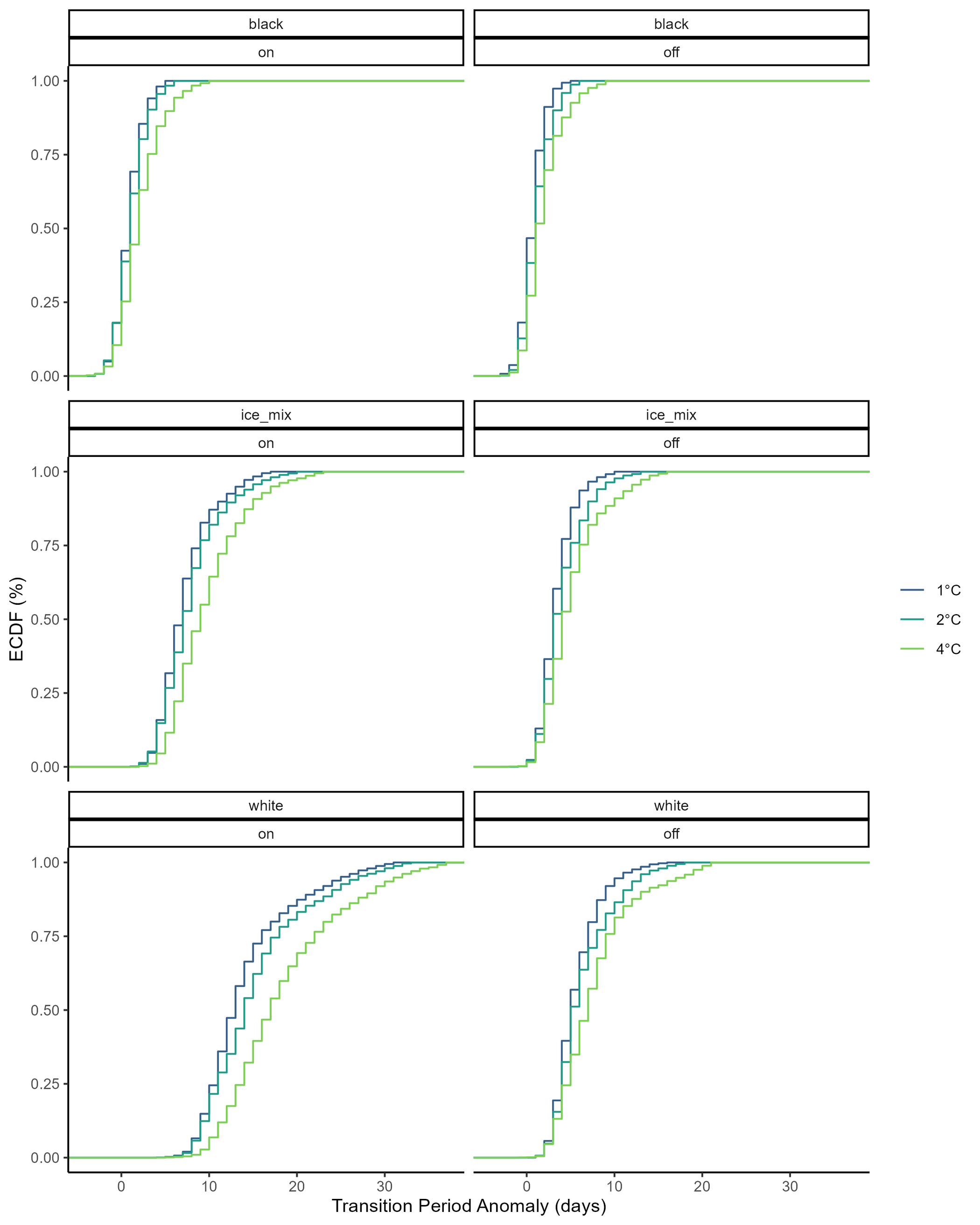

Supplement: S4 Fig — The same as in S3 Fig but represented are the ECDF of each ice quality scenario (100% black ice, 50% black ice– 50% white ice, and 100% white ice) with warming held constant. (TIF) [file pone.0313994.s004.tif]
