# Supplementary material for: Widespread loss of safe lake ice access in response to a warming climate
Source: PLoS One. 2024 Dec 11;19(12):e0313994. doi: 10.1371/journal.pone.0313994 (PMC11633986; doi:10.1371/journal.pone.0313994)
Supplement: S1 Table — The results of the Shapiro-Wilks test for each ice quality and warming scenario. (PDF) [file pone.0313994.s005.pdf]

S1 Table. Shapiro-Wilks Result Table.

Test	Transition Period	Ice type	Warming	p	n
**Shapiro-Wilks**	Formation	100% black	1 °C	<0.05	2734
			2 °C	<0.05	2641
			3 °C	<0.05	2529
		50% black/white	1 °C	<0.05	2616
			2 °C	<0.05	2559
			4 °C	<0.05	2404
		100% white	1 °C	<0.05	2537
			2 °C	<0.05	2464
			4 °C	<0.05	2258
	Melt	100% black	1 °C	<0.05	2742
			2 °C	<0.05	2665
			3 °C	<0.05	2505
		50% black/white	1 °C	<0.05	2638
			2 °C	<0.05	2568
			4 °C	<0.05	2405
		100% white	1 °C	<0.05	2559
			2 °C	<0.05	2494
			4 °C	<0.05	2259

The results of the Shapiro-Wilks test for each ice quality and warming scenario.
