# Supplementary material for: Widespread loss of safe lake ice access in response to a warming climate
Source: PLoS One. 2024 Dec 11;19(12):e0313994. doi: 10.1371/journal.pone.0313994 (PMC11633986; doi:10.1371/journal.pone.0313994)
Supplement: S2 Table — The results of the Kruskal-Wallis rank sum test, which compared the ice formation and melt transition period anomalies by the lake ice quality scenario (i.e., 100% black ice, 50%black/white ice, 100% white ice). (PDF) [file pone.0313994.s006.pdf]

S2 Table. Kruskal-Wallis Results Table Comparing Quality Scenarios.

**Test**	**Transition period**	**Ice type**	**p**	**n**
**Kruskal-Wallis**	Formation	100% black	<0.05	7904
		50% black/white	<0.05	7579
		100% white	<0.05	7259
	Melt	100% black	<0.05	7912
		50% black/white	<0.05	7611
		100% white	<0.05	7312

The results of the Kruskal-Wallis rank sum test, which compared the ice formation and melt transition period anomalies by the lake ice quality scenario (i.e., 100% black ice, 50%black/white ice, 100% white ice).
