# Supplementary material for: Widespread loss of safe lake ice access in response to a warming climate
Source: PLoS One. 2024 Dec 11;19(12):e0313994. doi: 10.1371/journal.pone.0313994 (PMC11633986; doi:10.1371/journal.pone.0313994)
Supplement: S3 Table — The results of the Kruskal-Wallis rank sum test, which compared the ice formation and melt transition period anomalies by the warming scenario (i.e., 1°C, 2°C, 4°C). (PDF) [file pone.0313994.s007.pdf]

S3 Table. Kruskal-Wallis Results Table Comparing Warming Scenarios.

Test	Transition period	Warming scenario	p	n
**Kruskal-Wallis**	Formation	1 °C	<0.05	7887
		2 °C	<0.05	7664
		4 °C	<0.05	7191
	Melt	1 °C	<0.05	7939
		2 °C	<0.05	7727
		4 °C	<0.05	7169

The results of the Kruskal-Wallis rank sum test, which compared the ice formation and melt transition period anomalies by the warming scenario (i.e., 1 °C, 2 °C, 4 °C).
