# Supplementary material for: Widespread loss of safe lake ice access in response to a warming climate
Source: PLoS One. 2024 Dec 11;19(12):e0313994. doi: 10.1371/journal.pone.0313994 (PMC11633986; doi:10.1371/journal.pone.0313994)
Supplement: S4 Table — The results from the Dunn’s test of multiple comparison using rank sums within each ice group (black, white, etc.) across warming scenarios (i.e., 1°C, 2°C, 4°C). The adjusted p value is provided, using the Holm method for detecting the family-wise error rate. (PDF) [file pone.0313994.s008.pdf]

S4 Table. Comparing across Ice Quality and Warming Scenarios with Dunn's Test.

Test	Transition period	Ice quality	Comparison	Adjusted p	n
**Dunn's**	Formation	Black	1 °C - 2 °C	<0.05	5375
			1 °C - 4 °C	<0.05	5263
			2 °C - 4 °C	<0.05	5170
		50% white	1 °C - 2 °C	<0.05	5175
			1 °C - 4 °C	<0.05	5020
			2 °C - 4 °C	<0.05	4963
		100% white	1 °C - 2 °C	<0.05	5001
			1 °C - 4 °C	<0.05	4795
			2 °C - 4 °C	<0.05	4722
	Melt	Black	1 °C - 2 °C	<0.05	5407
			1 °C - 4 °C	<0.05	5247
			2 °C - 4 °C	<0.05	5170
		50% white	1 °C - 2 °C	<0.05	5206
			1 °C - 4 °C	<0.05	5043
			2 °C - 4 °C	<0.05	4973
		100% white	1 °C - 2 °C	<0.05	5053
			1 °C - 4 °C	<0.05	4818
			2 °C - 4 °C	<0.05	4753

The results from the Dunn's test of multiple comparison using rank sums within each ice group (black, white, etc.) across warming scenarios (i.e., 1 °C, 2 °C, 4 °C). The adjusted p value is provided, using the Holm method for detecting the family-wise error rate.
