# Supplementary material for: Widespread loss of safe lake ice access in response to a warming climate
Source: PLoS One. 2024 Dec 11;19(12):e0313994. doi: 10.1371/journal.pone.0313994 (PMC11633986; doi:10.1371/journal.pone.0313994)
Supplement: S7 Table — The results from the asymptotic two-sample Kolmogorov-Smirnov test. The compared samples are within the same warming scenario (i.e., 1°C, 2°C, 4°C) and across ice quality scenarios (i.e., 100% black ice, 100% white ice, and 50% white ice). (PDF) [file pone.0313994.s011.pdf]

S7 Table. Comparing Ice Quality across Warming Categories.

Test	Transition period	Warming scenaio	Comparison	Adjusted p	n
**Kolmogorov-Smirnov**	Formation	1 °C	Black – 50%	<0.05	5350
			Black - white	<0.05	5217
			50% - white	<0.05	5153
		2 °C	Black – 50%	<0.05	5200
			Black - white	<0.05	5105
			50% - white	<0.05	5023
		4 °C	Black – 50%	<0.05	4933
			Black - white	<0.05	4787
			50% - white	<0.05	4662
	Melt	1 °C	Black – 50%	<0.05	5380
			Black - white	<0.05	5301
			50% - white	<0.05	5197
		2 °C	Black – 50%	<0.05	5233
			Black - white	<0.05	5159
			50% - white	<0.05	5062
		4 °C	Black – 50%	<0.05	4910
			Black - white	<0.05	4764
			50% - white	<0.05	4664

The results from the asymptotic two-sample Kolmogorov-Smirnov test. The compared samples are within the same warming scenario (i.e., 1 °C, 2 °C, 4 °C) and across ice quality scenarios (i.e., 100% black ice, 100% white ice, and 50% white ice).
