# Supplementary material for: Widespread loss of safe lake ice access in response to a warming climate
Source: PLoS One. 2024 Dec 11;19(12):e0313994. doi: 10.1371/journal.pone.0313994 (PMC11633986; doi:10.1371/journal.pone.0313994)
Supplement: Attachment — Submitted filename: Response to Reviewers.docx [file pone.0313994.s012.docx]

# Editor Comments:

**Comment:** *The manuscript has been reviewed by two anonymous reviewers. The study carry out a significant step forward in predicting lake ice safety in a warming climate, with important findings on how ice quality impacts safety and how warming affects the duration of safe and unsafe ice periods.*

**Response:** Thank you for considering our manuscript publication and the helpful revision summary. Please, see the individual comments and marked document for all of the responses and changes to the initial manuscript submission.

**Comment:** *However, some improvements are required such as terminology needs to be more precise, with clearer definitions for key terms.*

**Response:** Using the reviewers’ suggestions, we have added important context to the introduction and we have ensured that key terms are clearly defined throughout the manuscript.

**Comment:** *The abstract and introduction should provide more context and move detailed climatic information from the discussion to the introduction.*

**Response:** We have worked to add the needed context for the abstract and introduction. The specific changes can be seen in the responses to the reviewers.

**Comment:** *Model validation should be expanded with more detailed statistical analysis and inclusion of validation figures in the main text.*

**Response:** We agree and have expanded the validation to now include 71 ice thickness records between 1980-present. We found that the RMSE is 18 cm and the R^2^ = 0.74, where the modeled data tend to underestimate the observed peak ice thickness, but the ice growth is predicted within one standard deviation of the model mean. We added a figure comparing both the modeled and observed lake ice thickness for all lakes in the dataset alongside a time series that shows the variation between the modeled and observed data for one of the studied lakes. For more details and added text, please, see the responses to reviewers 1 and 2.

**Comment:** *The results and discussion sections need deeper exploration of model predictions and drivers of variability. I hope the revision will strengthen the quality, readability, precision, and validation.*

**Response:** We have worked to revise these sections with the feedback from reviewers 1 and 2. We offer additional information within the results section on spatial variability and its drivers as well as more discussion to synthesize these results in section 4.1.

# Reviewer #1:

**Comment:** *This paper addresses an important and interesting problem: lake ice phenology and quality to better assess safe lake ice use during the formation and melt periods.*

**Response:** Thank you for noting the importance of this manuscript dealing with what we see as a critical component of lake ice and cryosphere studies.

**Comment:** *1) This paper described that “Lake ice on timing was validated against 328 lakes from the Global Lake and River Ice Phenology database with >20 years of data (20). The correlation coefficient r was 0.78 with a mean absolute error (MAE) of 11.7 days. Ice-off timing had an r = 0.94 with an MAE of 15.6 days from a sample of 322 lakes. Ice thickness was validated with records from 15 Canadian lakes with an r = 98 0.93”.*

*(1) it needs to provide detailed information about spatio-temporal distribution of lake ice-on and ice-off timing and lake ice thickness.*

**Response:** We have indeed additional context to the introduction regarding lake ice timing and thickness. We have also added a section to the results that describes the spatio-temporal distribution of transition periods. We added the following to better describe these aspects of the results. You can also view our response to reviewer 2’s inquiry regarding the spatial variability of grids.

“Transition periods show distinct regional variability throughout the Northern Hemisphere but overwhelmingly shift toward longer transition periods. Unsafe transition periods on average increased (Table 1; Figure 3) and transition periods tended to increase the most along coastal regions in North America as well as southern latitudes in North America, Europe, and Asia. For example, transition periods exceeding 10 days composed approximately 30% of grids (n = 661) for white ice conditions at 4 °C. On the other hand, grids shifting to longer transition periods consisted of less than 1% of grids (n = 14). More grids shift toward longer transition periods at 1 C under black ice conditions (18%, n = 496). These grids are primarily located in northern latitudes of North America, Europe, and Siberia as well as some grids along the western coast of Greenland.”

*(2) The correlation coefficient r was 0.78 with a mean absolute error (MAE) of 11.7 days. Ice-off timing had an r = 0.94 with an MAE of 15.6 days from a sample of 322 lakes. There are a large uncertainties of research results like “… The transition period of unsafe ice increases by 4.97 ± 3.67 days in a 4 °C warmer world…”,” …The unsafe transition 33 period increases by an average of 19.8 ± 8.84 days and 8.75 ± 6.63 days…” according to MAE (Mean Absolute Error). How to proof the accuracy of results?*

**Response:** We have significantly revised the methods section to more accurately tailor to the research that we conducted. Initially, we highlighted the validation work conducted in Huang et al., 2022 who conducted analyses regarding lake ice thickness. We have revised the methods to focus on lake ice thickness results by increasing the validation lakes from 15 to 71. We included the following to the methods section to fully describe the validation process:

“The spatial extent and resolution of the study include the Northern Hemisphere on a 0.9°-by-1.25° gridded scale. Within each grid, the LISSS coupled to the CESM2-LE uses a sample lake to model ice thickness, where the sample lake has a depth and surface area of the mean of individual lakes within the grid, taken from the Global Lake and Wetland Database (22) and global gridded depth data at a 1 km resolution (23). CESM2-LE offers data for lake ice-on and ice-off timing, using the daily lake ice thickness from the one-dimensional lake ice model. The LISSS model has shown accurate representation of ice thickness and other variables (e.g., water temperature and surface fluxes) in previous studies (16,20,24–27). Ice thickness was validated with records from 15 Canadian lakes with an R^2^ = 0.86. While the correlation coefficient was high, the modeled data deviated from the 1:1 line, such that the model tended to underpredict ice thickness (slope = 2.1) (16). This bias toward thinner ice cover values is likely due to the warm bias of the simulated land surface air temperature in the fully coupled model, which was warmer than the ERA5 reanalysis data between the study years 1981-2020 and 30°N-70°N latitude by 1.5 °C (16). Additional validation information can be found in (16) and the associated supplementary information.

Owing to the small sample size in (16), we sought to further validate the ice thickness component of LISSS. We aggregated 71 lakes from datasets from Finland (28), North America (29,30), and Russia (31). Using 71 ice thickness records with data between 1980-present, ice thickness has a root mean square error (RMSE) of 18.5 cm and linear regression shows an R^2^ = 0.74 (n = 71) (Figure 1). As with the validation procedure in (16), the modeled data tended to underpredict ice thickness, which is evident from the slope of 0.49 (Figure 1a). When looking directly at lake ice thickness time series, the mean ice thickness of the ensemble members tends to underestimate the peak ice thickness (Figure 1b). The mean and standard deviation may also underpredict the peak ice thickness; however, they tend to capture the ice growth period and ice loss period, which are most important for this study. The model also tends to predict lake ice every year (1980-2024); however, some lakes did not freeze in years during data collection. It should be noted that the lake ice thickness model (LISSS) uses a mean lake area and depth to model ice thickness (16,20). Therefore, peak lake ice thickness is likely to vary between lakes that do not approximate that mean area and depth. ”

**Comment:** *2) The introduction could be enhanced by adding relevant studies on the state of lake ice cover in the Northern Hemisphere, and even globally, under the background of climate warming. This would provide some research background support for this study.*
**Response:** We revised the first paragraph of the introduction to add context regarding the state of lake ice cover in the Northern Hemisphere. The revised paragraph now reads:

“Anthropogenic climate change is causing rapid loss of lake ice in the Northern Hemisphere, driven largely by rising air temperatures (1). Studies have illustrated that the timing of ice-on and ice-off (ice phenology) is changing around the Northern Hemisphere. Lake ice duration has been a central concern, where long-term records have shown an overall increase in open-water days of 0.62 days per decade between 1931-2005, with a nonlinear increase in the rate of open-water days in the last 30 years of the time series (2). Lake ice phenology through long-term records extending over a century (1846-2019) show ice formation to be 11 days later and ice breakup to be 6.8 days earlier, which was a near doubling of ice loss compared to the same lake ice records between 1846-1995 (3). Therefore, long-term lake ice records show an overall loss of ice cover duration, timing, and thickness directly related to warming air temperatures (1,4).”

**Comment:** 3) *It is recommended to add a discussion section about the prospects of the paper, discussing whether future research could propose opinions on regional lake ice safety based on ice thickness. For example, which areas have feasible ice safety and which do not.*
**Response:** We have added a discussion section to address the prospects of the paper. We focus on areas that are of highest concern for lake ice safety as well as why certain regions may no longer have safe ice in the future. We have added the following to discussion section 4.3.

“The loss of safe lake ice conditions is a distinct reality for regions of the Northern Hemisphere. Our results indicate that lake ice will no longer be safe in more southern latitudes where lake ice may no longer grow thick enough for safe use in a warming world, especially when lake ice quality degrades and requires a thicker ice column to support human weight (5). Lake ice is likely to become less consistent with warming air temperatures throughout the 21st Century, leading to intermittent ice cover in southern latitudes (67). Moreover, as many as 5,700 lakes may also permanently lose ice cover with warming throughout the century (68). Research using multiple model means, including the Community Land Model 4.5 (CLIM4.5), projects that lake ice thickness will decline by 3.3 cm per degree Celsius increase in air temperature (1). An average loss of approximately 13.2 cm of ice thickness will make lakes at southern latitudes and coastal areas more likely to become unsafe, as their ice thickness is generally lower than northern latitudes (4). These regions, therefore, should be particularly sensitive to the effects that changing ice thickness and quality have on human safety as the world warms.”

**Comment:** *4) It is suggested to divide the methods and results sections into paragraphs, such as making the methods the second section (2. Methods, 2.1, 2.2, 2.3, etc.), to make the research process clearer and easier for readers to understand. Adding a graphical abstract would be even better.*

**Response:** We have included the section titles as recommended throughout the document for each section.

# Reviewer #2:

**Comment:** *This manuscript presents a model study of lake ice phenology and quality changes associated with warmer air temperatures. It represents a critical step forward toward predicting the safety of lake ice in a warmer world. The model predictions indicate that in addition to shorter ice cover periods, the period from unsafe ice to safe ice is lengthening. An important result is that ice quality can be the dominant determinant of ice safety. However, I find that some of the terms could be defined more precisely, and model validation could be improved.*

**Response:** Thank you for your brief summation of the paper, which helps confirm that the key points were accessible. We also thank you for the thorough review, including the more precise defining of terms and enhancing the model validation.

## General comments:

### Abstract:

**Comment:** *Please include a brief explanation of how ice quality impacts ice safety.*

**Response:** We appreciate that a brief explanation of ice quality and safety can help the impact of the abstract. We have added the sentence, “Later formation and earlier breakup of lake ice lead to overall short periods of use, but greater proportions of white ice, which may inhibit safe ice use due to its lower weight-bearing capacity.”

**Comment:** *Please describe briefly the analysis to contextualize the changes in the transition period. For example, the scope of the analysis is unclear; how many lakes (or grid cells with lakes) were modelled?*

**Response:** We used the Community Earth System Model version 2 Large Ensemble (CESM2-LE) to calculate the period when ice first appears until it is of sufficient thickness for safe use, depending on the ratio of black to white ice. We conducted this analysis for between 2,379-2,829 ~1°x1° grids throughout the Northern Hemisphere. We added the following to the abstract: “We used the Community Earth System Model Version 2 Large Ensemble (CESM2-LE) to calculate the period when ice first appears until it is of sufficient thickness for safe use, which depends on the ratio of black to white ice. We conducted this analysis ranging from 2,379 to 4,829 ~1° by 1° grid cells throughout the Northern Hemisphere. We focus on the period between ice formation (≥ 2 cm) to a safe thickness for general human use (i.e., ≥10, ≥15, or ≥20 cm, depending on the ratio of black to white ice).”

**Comment:** *The language is vague in places: ‘up to 35 days longer in a warming world’. What does ‘a warming world’ mean quantitatively?*

**Response:** We chose to include both the low (black ice) and high (white ice) transition period boundaries for the average increases in the transition periods rather than just the white ice conditions. We also added the specific temperature increase that led to those values.

We have amended the sentence to read, “We show that although many lakes are forecasted to freeze, they will be unsafe to use for between 5 and 29 fewer days in a 4 °C warmer world for black and white ice conditions, respectively.”

### Introduction:

**Comment:** *I miss a paragraph explaining the formation and properties of black versus white ice. Why is white ice considered less safe?*

**Response:** Please see the next comment for the full revision of the introductory paragraphs.

**Comment:** *I also miss a paragraph briefly describing the anticipated or ongoing climatic changes that affect lake ice formation, such as rising winter air temperatures, changing wind speed and a shift in the type and the amount of precipitation. OK, I see now that some of this information is included in the Discussion (L276-289). I would recommend moving this paragraph to the introduction.*

**Response:** We agree that paragraphs explaining the formation and properties of black versus white ice (comment above) and how climatic changes will help better contextualize the research as a whole. We have revised the introduction to include a discussion of the current condition of lake ice broadly (based on a comment from Reviewer #1) and how ice quality is important, how it develops, and how it may change. The first two paragraphs of the introduction have been revised as such:

“Anthropogenic climate change is causing rapid loss of lake ice in the Northern Hemisphere, driven largely by rising air temperatures (1). A multitude of studies has illustrated that the timing of ice-on and ice-off (ice phenology) is changing around the Northern Hemisphere. Lake ice duration has been a central concern, where long-term records have shown an overall increase in open-water days of 0.62 days per decade between 1931-2005, with a nonlinear increase in the rate of open-water days in the last 30 years of the time series (2). Lake ice phenology through long-term records extending over a century (1846-2019) show ice formation to be 11 days later and ice breakup to be 6.8 days earlier, which was a near doubling of ice loss compared to the same lake ice records between 1846-1995 (3). Therefore, long-term lake ice records show an overall loss of ice cover duration, timing, and thickness directly related to warming air temperatures (1,4).

However, climate change and warming winters have farther reaching effects by degrading lake ice quality conditions (7,8). Ice quality depends in large part on winter air temperatures, where warm temperatures induce diel melt-freeze cycles and/or precipitation to fall as both snow or rain. These temperature and precipitation conditions lead to white ice formation from freezing slush that introduces many air bubbles and small ice crystals that reflect light (9,10). White ice is also notable for being structurally weaker than black ice (11). The closer to 0 °C the air temperature is, the weaker the ice becomes (12). For example, white ice at -0.5 °C is 51% weaker than black ice at the same temperature, owing to the increased strength of ice with increased density (12). White ice formation is generally expected toward the end of the season when temperatures warm and snow atop the ice column leads to a larger fraction of white ice as the ice column evolves throughout the season toward breakup (7). However, white ice conditions are possible at the beginning of the season as well when ice formation and snow conditions coincide to form larger proportions of white ice (13). Therefore, warming conditions expected throughout the 21st Century may lead to a more consistent presence of white ice throughout the season.”

### Methods:

**Comment:** *If I understand correctly, the authors used the CESM2-LE + LISSS model to obtain predictions of ice thickness at various climate warming scenarios, and then computed the length of the ‘unsafe’ and ‘safe’ ice periods based on ice thickness recommendations from the MDNR and the assumption that the ice is either 100% black ice, 50% black ice and 50% white ice, or 100% white ice.*

**Response:** Yes, that is the correct interpretation.

**Comment:** *Outcomes of statistical tests should include the analysis and the sample size (e.g. L96, r = 0.92).*

**Response:** We originally included the statistical test and removed the sample size from the sentence and placed it within a parenthetical after the coefficient of determination for ease of reading. These sentences read:

“CESM2-LE offers data for lake ice-on and ice-off timing, using the daily lake ice thickness from the one-dimensional lake ice model. Lake ice on timing was validated against 328 lakes from the Global Lake and River Ice Phenology database with >20 years of data (25). The correlation coefficient R^2^ = 0.61 (linear least-squares regression, n = 328) with a mean absolute error (MAE) of 11.7 days. Ice-off timing had an R^2^ = 0.88 (linear least-squares regression, n = 322) with an MAE of 15.6 days. Ice duration had an R^2^ = 0.88 (linear least-squares regression, n = 320) with a MAE of 17.3 days. Ice thickness was validated with records from 15 Canadian lakes with an R^2^ = 0.86.”

However, we have replaced the description of the ice phenology validation from Huang et al., 2022 with a validation of ice thickness where we expanded the number of lakes from 15 to 71. Please, see the comment below for a full description.

**Comment:** *Why is the Pearson correlation coefficient (r) used here and not the coefficient of determination (r2), which is usually used to determine model fit?*

**Response:** Originally, we decided to record the Pearson correlation as in the original validation material found in the supplemental material of Huang et al., 2022 (DOI: 10.1038/s41467-022-33495-3). However, we have updated the values to the coefficient of determination as you have advised along with the changes laid out in the previous comment. Please, see the previous comment for the updated text.

**Comment:** *The model validation could be expanded upon. For example, you can have a high coefficient of determination, but a significant deviation from the 1:1 line. I suggest that figures showing the model validation be included in the main text. I would also suggest that the authors look for a larger and more representative lake ice thickness dataset to validate the model.*

**Response:** You are correct. In the validation in Huang et al., 2022, which we cited in the methods, the coefficient of determination was high, but the slope deviated from the 1:1 line. This result shows that the model underestimates ice thickness, owing to a warm bias in CESM2-LE. We have expanded on the initial validation by adding additional lakes with data between 1980-2024. We have also included a figure (figure 1a,b) to visualize the discrepancy between the modeled and observed data.

We have added the following to the methods:

“The spatial extent and resolution of the study include the Northern Hemisphere on a 0.9°-by-1.25° gridded scale. Within each grid, the LISSS coupled to the CESM2-LE uses a sample lake to model ice thickness, where the sample lake has a depth and surface area of the mean of individual lakes within the grid, taken from the Global Lake and Wetland Database (22) and global gridded depth data at a 1 km resolution (23). CESM2-LE offers data for lake ice-on and ice-off timing, using the daily lake ice thickness from the one-dimensional lake ice model. The LISSS model has shown accurate representation of ice thickness and other variables (e.g., water temperature and surface fluxes) in previous studies (16,20,24–27). Ice thickness was validated with records from 15 Canadian lakes with an R^2^ = 0.86. While the correlation coefficient was high, the modeled data deviated from the 1:1 line, such that the model tended to underpredict ice thickness (slope = 2.1) (16). This bias toward thinner ice cover values is likely due to the warm bias of the simulated land surface air temperature in the fully coupled model, which was warmer than the ERA5 reanalysis data between the study years 1981-2020 and 30°N-70°N latitude by 1.5 °C (16). Additional validation information can be found in (16) and the associated supplementary information.

Owing to the small sample size in (16), we sought to further validate the ice thickness component of LISSS. We aggregated 71 lakes from datasets from Finland (28), North America (29,30), and Russia (31). Using 71 ice thickness records with data between 1980-present, ice thickness has a root mean square error (RMSE) of 18.5 cm and linear regression shows an r2 = 0.74 (n = 71) (Figure 1). As with the validation procedure in (16), the modeled data tended to underpredict ice thickness, which is evident from the slope of 0.49 (Figure 1a). When looking directly at lake ice thickness time series, the mean ice thickness of the ensemble members tends to underestimate the peak ice thickness (Figure 1b). The mean and standard deviation may also underpredict the peak ice thickness; however, they tend to capture the ice growth period and ice loss period, which are most important for this study. The model also tends to predict lake ice every year (1980-2024); however, some lakes did not freeze in years during data collection. It should be noted that the lake ice thickness model (LISSS) uses a mean lake area and depth to model ice thickness (16,20). Therefore, peak lake ice thickness is likely to vary between lakes that do not approximate that mean area and depth.”

**Along with the following figure:**

### Results

**Comment:** *A key part of the results is a comparison between modelled transition times from ice formation to ‘safe’ ice for both black ice and white ice. How realistic is the immediate formation of white ice after ice-on, given what we know about the formation process of white ice? For example, does the model predict more freeze-thaw cycles at the start of the season? If it is not a realistic scenario, is it useful?*

**Response:** While complete black or white ice conditions are not realistic for all lakes, it felt essential to offer the full range of potential transition periods, given 100% black ice to 100% white ice conditions. We agree that the formation of complete white ice at the start of the season is unlikely to happen consistently. White ice does tend to grow as “superimposed” ice on top of primary and secondary ice (Michel & Ramseier, 1971, DOI: 10.1139/t71-004). White ice can be the initial layer of lake ice in instances of pancake ice and agglomerated ic, though those instances tend to rely on large lakes with long fetches that permit wind to break up primary and initial secondary ice.

However, it has been noted by Weyhenmeyer et al., 2022 that white ice can make up the entire ice column shortly after formation during warm years, as the IceBlitz project found in the winter of 2020 in several lakes in North America and Europe, which led to generally unsafe ice conditions during sampling. Early white ice formation is also present in areas that experience early winter snows as well as free and thaw cycles that lead to large portions of white ice in the ice column, as in Socha et al., 2023 (DOI: 10.1002/lno.12319).

For these reasons, we chose to include the full breadth of values, because many areas are likely to experience high proportions of white ice early in the season, particularly in warming climates.

**Comment:** *The regional distribution of transition times is not mentioned in the results, but it appears that some regions are transitioning to longer ‘safe’ ice seasons, correct?*

**Response:** Yes, there are some regions that are transitioning to longer safe ice periods. These regions are primarily in northern latitudes of North America, Europe, and Siberia. For example, at 1 °C and black ice, there are 496 grids that are shifting to longer transition periods. At 4 °C and black ice, there are 266 grids. On the other hand, for white ice conditions at 1 °C, there are 184 grids shifting to longer transition periods. By 4 °C, there are only 14 grids. The patterns are similar for longer transition periods, but the grids decrease dramatically, especially between 2 °C and 4 °C.

We have added the following paragraph to the results section that describes the spatial variability of the transition periods:

“Transition periods show distinct regional variability throughout the Northern Hemisphere but overwhelmingly shift toward longer transition periods. Unsafe transition periods on average increased (Table 1; Figure 3) and transition periods tended to increase the most along coastal regions in North America as well as southern latitudes in North America, Europe, and Asia. For example, transition periods exceeding 10 days composed approximately 30% of grids (n = 661) for white ice conditions at 4 °C. On the other hand, grids shifting to longer transition periods consisted of less than 1% of grids (n = 14). More grids shift toward longer transition periods at 1 C under black ice conditions (18%, n = 496). These grids are primarily located in northern latitudes of North America, Europe, and Siberia as well as some grids along the western coast of Greenland.”

### Discussion

**Comment:** *The discussion seems to be a combination of introduction and results. I’d like to see a deeper exploration as to why the model predicts the shifts in the transition times (Fig. 2) and what variable(s) determine(s) their global distribution (Fig. 3). Some of this information should be in the model results. For example, are the number of freeze-thaw cycles increasing? Is the decline in the ice growth rate due to the direct influence of warmer air temperatures or also indirect effects, such as increasing precipitation as snow (insulation) or rain (melt)?*

**Response:** We have significantly revised the discussion to reduce text that appears to be introductory material and results oriented material. The resulting discussion attempts to better place this research in the context of other lake ice phenology work. For example, see the response to the next comment.

**Comment:** *Figure 2 is not discussed in much detail. What are the drivers of the spatial variability in transition period trends?*

**Response:** We have significantly altered what is now section 4.1 which discusses the projected behavior of lake ice. We have added discussion of spatial variability and the influences of that variability as well as changes in transition period variability.

“Ice quality impacts the carrying capacity of lake ice, which interacts with air temperatures both in the creation of white ice (5,7) and its flexural strength (9,10). As density decreases with degrading ice quality, lake ice can support less weight and becomes more than 50% weaker at temperatures near 0 °C (10). Ice quality can quickly degrade in warm years, where most or all of the ice column can become white ice throughout the ice cover season, resulting in dangerous ice conditions, including drownings (5,15). Focus has been placed within lake ice research on ice phenology (i.e., timing) and ice duration (3,17,48), as well as ice thickness (1,4,16). While the duration of safe lake ice use has been analysed for transportation efforts (21), the unsafe transition period adds important context because people often use lake ice in the early winter and late spring for recreation, when a majority of incidents take place (15).

Like with ice phenology and duration, air temperature plays a large role in ice cover development and melt. Air temperature is the primary driver of lake ice formation, growth, and melt, and increasing global air temperatures will shift lake ice timing and diminish thickness (1,49,50). While the magnitude of the trends in ice phenology and duration will vary based on the study, data used, and model type, some of the most recent, long-term trends the current most comprehensive collection of global lake ice records, extending from 1846 to 2019, indicates that warming air temperatures have resulted in delayed ice formation of 11.0 days and advanced breakup of 6.8 days (39). Even while ice cover is diminishing in duration throughout the Northern Hemisphere, we found that warming lengthened the amount of time that ice cover is unsafe for use, even if it is present.

While ice cover is thickened by snow cover and lakes in regions with increased snow cover can show signs of increasing in thickness (51), we found that air temperature is the governing factor in the lengthening of transition periods. When comparing CESM2-LE modeled results for air temperature, snow depth, and lake ice thickness, we found that thickness is overwhelmingly affected by air temperature rather than snow cover (Figure S1). In areas throughout the Northern Hemisphere that are warming more, ice thickness followed that trend with decreasing ice thickness. The spatial patterns of unsafe transition period anomalies followed that pattern (Figure 3). Transition periods tended to increase most in areas where warming is expected, such as lower latitudes and coastal regions (1,16). In fact, our derived transition period anomalies followed similar spatial patterns to both studies that used CESM2-LE lake ice thickness data in other capacities (16,27) and those that used other methods to describe patterns in lake ice (1,52). Areas that experience longer transition periods are in the minority; however, these regions are likely experiencing cooler temperatures (6).

Finally, changes in transition variability will impact the predictability of safe ice conditions. Lake ice is typically safe in temperate Northern Hemisphere lakes in February when many people use lakes for recreation; however, accidents have occurred as lake ice becomes less predictable (5,15,50). Lakes are projected to enter no-analogue scenarios as early as 2050 where average metrics such as lake temperature exceed current variability (27). Additionally, lake ice duration has become more variable, especially when ice duration has significantly decreased (53). The increasing standard deviation when temperatures increase and ice quality degrades indicates that lakes may have increasingly unpredictable safety conditions during ice cover periods as durations decrease and ice quality degrades (5,53).”

## Specific comments:

**Comment:** *L29: doesn’t the safe ice thickness depend on the ratio of black and white ice?*

**Response:** We note that this phrasing is possibly a bit confusing. You are correct that safe ice thickness partially depends on the ratio of black and white ice. So we have amended the sentence to clarify the relationships between ice thickness and quality.

The sentence now reads, “We focus on the period between ice formation (≥ 2 cm) to a safe thickness for general human use (i.e.,≥ 10 cm , depending on the ratio of black to white ice).”

**Comment:** *L31-33. ‘4.97 ± 3.67 days’ given the confidence interval (standard deviation?) it may be more appropriate to use one decimal here.*

**Response:** We have amended the values to only include one decimal place. We have also made this change throughout the manuscript, including all results and tables.

**Comment:** *L36. ‘increase the risk of drowning’. Is this substantiated?*

**Response:** Yes, in the second paragraph of the introduction, we describe conditions under which compromised ice quality led to drownings in Sweden as recorded by Weyhenmeyer et al., 2022. However, as the abstract’s structure has changed through the review process, this sentence no longer appears in the abstract.

**Comment:** *L37-38: ‘when black ice transitions to white ice conditions’. Doesn’t this usually happen at the end of winter?*

**Response:** That is the typical progression but not a rule. A large percent of white ice can form at the beginning of the season, depending on meteorological conditions. To avoid confusion and reduce the word count of the abstract, we have altered the sentence to be more aligned with the previous change on lines 41-42.

The sentence now reads, “This wide range indicates that ice quality has a strong influence on ice safety.”

Therefore, it reinforces the variable lengths of the unsafe transition period under differing ice quality conditions, but does not give the example that you point out.

**Comment:** *L50: only North America?*

**Response:** Thank you for catching that mistake. This should read “Northern Hemisphere” rather than “North America.” We have amended the sentence.

**Comment:** *L57: what is meant by ‘stable’?*

**Response:** We appreciate that this term may be too vague. The intent was to show that black ice has more flexural strength than white ice; however, we wanted to avoid jargon to increase accessibility. To this end, we have replaced “less stable” with “weaker.”

**Comment:** L62. Here or earlier, please provide a working definition of ‘safe ice’ (e.g. safe for one person to walk on, 5 cm for black ice and 10 cm for white ice) – with a reference (e.g. ref. 7 or Gold 1971).

**Response:** *We agree that this addition will make the concept of “safe ice” or “unsafe transition periods” more transparent. After the sentence you have marked we added:*

“Safe ice conditions are defined by a combination of thickness and ice quality, where 10 cm of black ice or 20 cm of white ice are required for a person to safely walk on the ice (7).”

**Comment:** *L97-98: why were only 15 lakes used to validate lake ice thickness?*

**Response:** Initially, 15 lakes were used in the CESM2-LE lake ice thickness validation process carried out in Huang et al., 2022 (ref. 5). However, we have since included additional lake ice thickness data to increase the number of validation lakes. Please see Figure 1a and the revised validation section within the methods section for additional information. In short, there are now 71 lakes used for lake ice thickness validation with a RMSE of ~18 cm. The mean of the model ensemble members tends to underpredict the maximum lake ice thickness but represents the growth and decay of ice well (figure 1b).

**Comment:** L102: *What does ‘weaker’ mean here? Barrette (ref. 21) estimated the flexural strength of ice, which is proportional, but not identical to load-bearing strength. However, their results roughly correspond to those of Gold 1971, who found that the load-bearing capacity of white ice was about 50% that of black ice based on documented ice failure under vehicular loads.*

**Response:** We agree that “weaker” may be too vague. We have followed your advice and amended the sentence to read:

“For example, the flexural strength of white ice is approximately 51% lower than black ice at temperatures near 0 °C (21) and the load bearing capacity of lake ice can drop significantly when ice quality is low (22).”

**Comment:** *L108: please add that the ice quality gradients are based on assumptions/scenarios of 100% black ice, 50% black ice and 50% white ice, and 100% white ice.*

**Response:** We have added the following sentence to make this clarification:

“The ice quality gradients in this study focus on assumed scenarios of lake ice at 100% black ice, 100% white ice, and a 50% black ice and 50% white ice scenario.”

**Comment:** *L110: why is 2 cm used as the threshold for ice formation, and not >0 cm?*

**Response:** We agree that this is unintuitive without further context. We have added the following explanation:

“We chose 2 cm as ice-on to eliminate short-lived, intermittent ice events that might occur if defining ice as >0 cm. For example, the initial skim ice that forms on a calm, cold night can break up easily from wind stress or melt from warmer temperatures during the day (7). These periods of brief ice cover are not typically considered as the date of ice formation in ice phenology records and are known to add uncertainty to lake ice records (27). Using an ice thickness > 2 cm permitted us to consider the ice column of each lake within a grid to be stable. ”

**Comment:** L112: *What load (e.g. a person, or a car) do these safety recommendations apply to?*

**Response:** We have added the following explanation to describe the load:

“For pure black ice, a thickness of 10 cm was used to conform with the MDNR safety recommendations for a person walking on ice.”

**Comment:** *L117: Why these temperatures? Do they correspond to specific climate warming scenarios?*

**Response:** We have added additional context to the temperature thresholds. Following the sentence that you note, we have added:

“These temperature thresholds were chosen to cohere with an established framework of indexing global temperature rise instead of a particular emission scenario since lake ice cover is strongly influenced by the global mean surface temperature (25).”

**Comment:** *L119: were some grid cells excluded from the analysis? These could be grid cells without lakes or without freezing days. If so, please describe the selection procedure.*

**Response:** Yes, thank you for pointing out this omission. We excluded lakes that were frozen for very short and very long periods. We only included lakes that were frozen >30 days and <330 days. We added the following sentence to clarify:

“We also excluded grids where ice cover lasted for fewer than 30 days or longer than 330 days.”

**Comment:** *L121-122: Please provide a (supplementary) table showing the outcomes of all your statistical tests, including the test statistic and the sample size.*

**Response:** We have included the requested table in the supplementary material as Tables (i.e., Tables 1 - 3) with reference to the tables in the text.

**Comment:** *L121-122: ‘Shaprio-Wilks’ -> ‘Shapiro-Wilks’*

**Response:** Thank you for catching this and the following typos. We have corrected this in the text.

**Comment:** *L121-122: why is the Shapiro-Wilks test done when the other statistical tests are non-parametric (i.e. do not require the assumption of a particular distribution)?*

**Response:** We used the Shapiro-Wilks test to determine if the data deviated from a normal distribution. We found that the data did deviate from a normal distribution, so we proceeded with non-parametric statistical tests.

**Comment:** *L124: ‘Kurskal-Wallis’ -> ‘Kruskal-Wallis’*

**Response:** We have corrected this typo in the text.

**Comment:** *L124: conventionally (and arbitrarily), p < 0.05 is used. Why this threshold?*

**Response:** We noted that the p values met a lower threshold and so we included the lower threshold. However, to avoid any confusion, we have reverted the p values to p < 0.05.

**Comment:** *L126: ‘Kalmogorov-Smirnov’ -> ‘Kolmogorov-Smirnov (KS)’*

**Response:** We have corrected this typo in the text

**Comment:** *L128: is this a two-sample comparison or a multiple comparison? If the latter, it is necessary to adjust p for the false discovery rate using the Benjamini-Hochberg or equivalent procedure.*

**Response:** It is a multiple comparison Dunn’s test. The results of the test included the Holm’s (1979) progressive step-up procedure to test for family-wise error rate, which is built into the function used for the Dunn’s test (see: Ogle et al., (2023) and Dinno (2024) - both listed in the references section). We have included the information of the test within the Methods section, the table description with the results (Table S2c,d), as well as the adjusted p values. Unlike the KW & Dunn’s test, the KS test was a two-sample comparison. All tests, p values, and other information are now provided in the supplementary tables.

**Comment:** *L162-163: please define the uncertainty estimates (SD, 95% confidence interval) and the sample size (n = …).*

**Response:** We have included the uncertainty estimate and the sample size as a parenthetical after each mean and SD value in the text. We have also updated Table 1 to include the standard deviation as a separate column and included the sample size to avoid any ambiguity moving between the text and the Table.

**Comment:** *L165-166: suggests changing ‘(≥10 cm, ≥15 cm, and ≥20 cm)’ to ‘(≥10 cm, ≥15 cm, and ≥20 cm, depending on assumed ice quality)’.*

**Response:** We have included the suggested addition.

**Comment:** *L179: Table 1, please include the outcome of the statistical test indicating whether or not each mean is significantly different from 0 at a specified confidence level (e.g. p = 0.05), or show the p-values.*

**Response:** For this table, we did not test whether the mean values were different from 0. We did, however, include the supplementary tables, which show that the distributions are not normally distributed in supplementary table S1.

**Comment:** L193: mention that you are talking about 100% black ice here.

**Response:** Yes, this sentence was unclear without the additional context. We have expanded it to include all of the thresholds. The sentence now reads:

“The unsafe transition period during the lake ice melt occurs when lake ice thins to ≤10 cm (black ice), ≤15 cm (50% black ice and 50% white ice), and ≤20 cm (white ice) until ice breakup (≤2 cm)”

**Comment:** *L219: Figure 3 caption. when mentioning p-values it is customary to also name the statistical test and the sample size.*

**Response:** We have added the test and we have included a reference to the supplementary tables for both the KW and KS tests. We opted to reference the tables because this sentence is an overall summary, and multiple sample sizes may be confusing.

**Comment:** *L233-244: this reads as part of the introduction. No results are discussed here as far as I can tell.*

**Response:** We originally intended this to be a summary of the main goals of the paper, but we can see how this reads like it belongs more to the introduction than as a reorientation of the research that the we conducted within the paper.

We have revised the introductory paragraph of the Discussion to better establish it as a reorientation to the knowledge gaps and the research that was conducted.

“This research investigated how projected warming average air temperatures throughout the 21st Century will result in longer periods when lake ice cover is not safe for human use. Studies in winter limnology have primarily focused on changing phenology and duration (1) and decreasing ice thickness (1,2). Using the CESM2-LE, our projections suggest that lake ice will be unsafe for human use for a mean of approximately 5, 17, or 29 days longer per year for black ice, 50% white ice, and 100% white ice at 4 °C. We also found that ice quality increased the transition period of unsafe ice more than did increasing air temperatures alone. ”

**Comment:** *L232: ‘forecast’ is a term not usually applied to short-term rather than long-term model predictions. ‘Project’ or ‘Predict’ may be more appropriate here.*

**Response:** We agree that project is a more accurate word to use in this instance and we have made that change.

**Comment:** *L245: suggest changing ‘forecasted’ to ‘projected’.*

**Response:** We have changed “forecasted” to “projected.”

**Comment:** *L246: did you mean ‘the primary mechanism through which we understand changes in ice phenology and duration…’?*

**Response:** We can see that this sentence is a bit confusing. We have altered the wording to better clarify the intent. It now reads:

“Air temperature is the primary driver of lake ice formation, growth, and melt, and increasing global air temperatures will shift lake ice timing and diminish thickness.”

**Comment:** *L248: ‘some of the most recent long-term trends’: what is meant here? Trends since the year 2000?*

**Response:** To clarify this statement and put it within a grounded temporal context (so that future readers can understand the timing of these trends), we have changed the sentence to the following:

“While the magnitude of the trends in ice phenology and duration will vary based on the study, data used, and model type, the current most comprehensive collection of global lake ice records, extending from 1846 to 2019, indicates that warming air temperatures have resulted in delayed ice formation of 11.0 days and advanced breakup of 6.8 days.”

**Comment:** *L252: please provide a bit more context for these previous studies. ‘ice thickness declines’; ice thickness varies over the season, so is this the mean ice thickness? A 1C rise in air temperature; is this the annual mean air temperature? And did these previous studies cover the same lakes/area as this study?*

**Response:** We agree that these values could be confusing without additional context. So we have revised the sentence to make it apparent that the referenced study also uses the same CESM2-LE data.

The text now reads: “Our results add critical context to previous analyses that also used CESM2-LE modeled lake ice data, which show a decline in lake ice duration (5), such that ice duration declines at a rate of -9.7 days °C^-1^ and ice thickness declines at a rate of 0.033 meters per a 1 °C rise in global annual mean air temperature.”

**Comment:** *L252: days C−1 -> days °C−1*

**Response:** We have added the degree symbol.

**Comment:** *L253: ‘projections forecast’: please see my previous comment on the use of ‘forecast’.*

**Response:** We have altered the text to eliminate “forecast.”

***Comment:*** *L257-258: which scenarios are compared here? (e.g. historical formation of black ice vs a future formation of white ice at 4°C).*

**Response:** We intended to compare black ice to white ice, both at 4 °C. We can see that including different ice quality ratios and incorporating the standard deviation within the values was confusing, so to clarify, we have amended the sentence to the following to focus on differences in the transition period with warming alone:

“Notably, the transition from ice-on to safe human use could be up to 2.6 ± 3.7 days after ice formation with climate warming, compared to 1.1 ± 2.3 days historically when comparing black ice at 4 °C to black ice at 1 °C.”

**Comment:** *L260: 9 days earlier. Do you mean ‘9 days before ice-off’ compared to ‘4 days before ice-off historically’? If so, again, which scenario is this prediction associated with? Also, this is a result which should be in the ‘Results’ section.*

**Response:** We were originally looking at ice timing here, but because the rest of the manuscript is focused on the length of the transition period, this sentence is confusing. However, to your point regarding this reading as results, we have entirely removed the statement, as it was not required in the results section. Instead, we have revised this section (what is now section 4.1) to better synthesize the findings from the results. Please, see section 4.1 for the revisions. We will not paste the text here, as the text is not a direct response to this particular comment.

**Comment:** *L276-289: this paragraph should be in the introduction. No results are discussed here.*

**Response:** We have relocated this content to the introduction and incorporated it into the text as suggested in the comment regarding the need for ice quality definitions in the introduction.

**Comment:** *L290: please provide references for the statements ‘rising winter and spring air temperatures’ and ‘increasing proportions of white ice’.*

**Response:** We have included references to these statements.

**Comment:** *L299: this was already stated on L297.*

**Response:** Thank you for catching that instance of repetition. We have deleted that sentence to eliminate the repetition.

**Comment:** *L304-305: is the 29 days an average for all the lakes at 4 °C? If so, is this the same modelling approach with the same lakes (i.e. are the results comparable)?*

**Response:** While Grant et al., 2021 use CLIM which is the land model used in CESM2-LE, the values derive from a multi-model mean, so the results are not as readily comparable as a study that used CESM2-LE. We have altered the text to reflect this.

“While the Northern Hemisphere as a whole will tend toward longer average transition periods (see Table 1), lakes may have extremely long transition periods (i.e., longer than 1 month), shortening the viable ice cover period for activities more than the 38 ± 11 days predicted from (16) for a warming by the end of the 21st Century using CESM2-LE ice thickness data.”

**Comment:** *L310: can the cumulative distributions be shown in a (supplementary) figure?*

**Response:** Yes, the cumulative distributions are now shown as supplementary figure 1a,b**.**

**Comment:** *L317: ‘increasingly unpredictable ice safety’. This is an important result. Could this be quantified? E.g. compare the standard deviation of the transition period length (not the anomaly) between historic and warming scenarios and see if it increases.*

**Response:** To quantify the increasing variability of the unsafe transition period, we have quantified the standard deviation, as you described. We included a table (Table 2) with those values, showing the increase in variability with increasing warming and degrading ice quality. We have also added the following text to the results section:

“Additionally, the standard deviation of the transition period increases with warming scenarios and across ice quality ratios, which implies that the variability of the transition period increases with both warming and degrading ice quality (Table 2). For example, the standard deviation of black ice during the ice formation period increases from 11.5 to 13.3 days between the historical period and 4 °C. The standard deviation of white ice, on the other hand, increases from 23.7 to 30.0 days between the same period. The standard deviation during the melt period is overall shorter compared to the ice formation period; however, the values increase with both warming and degrading ice quality (Table 2).”

**Comment:** *L346: this paragraph is a combination of methods and results. Please restructure.*

**Response:** The field work mentioned was based on research conducted first-hand, but currently is not substantive enough to include in full in either the methods and results section. Therefore, we have decided to remove this paragraph.

**Comment:** *L392: ‘which some individuals may fail to take into account’. This is unscientific speculation, please remove.*

**Response:** We agree that this is speculation and have removed the statement.
